# Harmonic Scalpel versus Electrocautery Dissection in Modified Radical Mastectomy for Breast Cancer: A Meta-Analysis

**DOI:** 10.1371/journal.pone.0142271

**Published:** 2015-11-06

**Authors:** Jinbo Huang, Yinghua Yu, Changyuan Wei, Qinghong Qin, Qinguo Mo, Weiping Yang

**Affiliations:** 1 The Department of Breast Surgery, Affiliated Tumor Hospital of Guangxi Medical University, Nanning, Guangxi, People’s Republic of China; 2 The Department of Ultrasound Diagnosis, Affiliated Tumor Hospital of Guangxi Medical University, Nanning, Guangxi, People’s Republic of China; University Medical Center of Princeton/Rutgers Robert Wood Johnson Medical School, UNITED STATES

## Abstract

**Background:**

Despite the common use of conventional electrocautery in modified radical mastectomy for breast cancer, the harmonic scalpel is recently emerging as a dominant surgical instrument for dissection and haemostasis, which is thought to reduce the morbidity, such as seroma and blood loss. But the results of published trials are inconsistent. So we made the meta-analysis to assess the intraoperative and postoperative endpoints among women undergoing modified radical mastectomy with harmonic scalpel or electrocautery.

**Methods:**

A comprehensive literature search of case-control studies from PubMed, MEDLINE, EMBASE and Cochrane Library databases involving modified radical mastectomy with harmonic scalpel or electrocautery was performed. We carried out a meta-analysis of primary endpoints including postoperative drainage, seroma development, intraoperative blood loss and secondly endpoints including operative time and wound complications. We used odds ratios (ORs) with 95% confidence intervals (CIs) to evaluate the effect size for categorical outcomes and standardised mean differences (SMDs) for continuous outcomes.

**Results:**

A total of 11 studies with 702 patients were included for this meta-analysis. There was significant difference in total postoperative drainage (SMD: -0.74 [95%CI: -1.31, -0.16]; *P*< 0.01), seroma development[OR: 0.49 (0.34, 0.70); *P* < 0.01], intraoperative blood loss(SMD: -1.14 [95%CI: -1.81,-0.47]; *P* < 0.01) and wound complications [OR: 0.38 (0.24, 0.59); *P* < 0.01] between harmonic scalpel dissection and standard electrocautery in modified radical mastectomy for breast cancer. No difference was found as for operative time between harmonic scalpel dissection and standard electrocautery (SMD: 0.04 [95%CI: -0.41, 0.50]; *P* = 0.85).

**Conclusion:**

Compared to standard electrocautery, harmonic scalpel dissection presents significant advantages in decreasing postoperative drainage, seroma development, intraoperative blood loss and wound complications in modified radical mastectomy for breast cancer, without increasing operative time. Harmonic scalpel can be recommended as a preferential surgical instrument in modified radical mastectomy.

## Introduction

Breast cancer is the most common malignancy and the major cause of cancer-related death with 23% of new cases and 14% of total deaths globally in female population around the world [1]. The incidence of breast cancer in developing countries, especially in China, has a significant increase these years due to the changed life-style [2]. Though breast-conserving surgery is more and more welcomed among female patients, modified radical mastectomy (MRM) still plays an important role in the operation for breast cancer.

Compared to conventional scalpel, electrocautery is the most common surgical instrument for dissection and haemostasis in MRM, with the advantage of reducing blood loss [3]. However, previous studies indicated that it may increase the risk of postoperative complications, such as seroma, wound infection, flap necrosis, hematoma, and prolonged drainage, which led to a delay of adjuvant treatments after the operation[4,5].

The harmonic scalpel, which is widely used in laparoscopic surgery, nowadays presents an encouraging prospect for dissection in MRM. The high frequency mechanical vibrations of harmonic scalpel make intraoperative cutting and coagulation take place at the same time, with a relatively low temperature causing fewer thermal injuries than electrocautery[6].

Some studies showed that the harmonic scalpel could shorten the dissection time and decrease blood loss, drainage volume, seroma development and wound complications as compared to electrocautery[7,8], while other papers indicated that neither clinical advantages nor disadvantages of the ultrasound dissection technique were found[9,10]. A previous meta-analysis implicated that harmonic scalpel dissection and standard electrocautery appeared to deliver similar results in the mastectomy setting[11]. However, newly published trial preferred harmonic scalpel as the instrument for its superiority in reducing postoperative discomfort and morbidity to the patient without increasing operating time[12]. Despite of the advantages of harmonic scalpel, the inconsistent results of published trials invite more attention to its usage in MRM. Therefore, we performed the meta-analysis including the latest data to evaluate the operative endpoints of patients undergoing MRM for breast cancer between harmonic scalpel and electrocautery.

## Methods

### Search strategy and selection criteria

A comprehensive literature search was performed by J.-B. Huang and Y.-H. Yu for studies up to June 22, 2015 in PubMed, Medline, Embase, and the Cochrane Library databases using the terms“diathermy” or “electrocautery” or “electrocoagulation” plus “ultrasonic” or “ultrasonics” or “ultracision” or “harmonic scalpel” in combination with“mastectomy”or“mammectomy”. We screened the reference lists of included studies to identify further related publications. The compared studies on patients who underwent MRM with harmonic scalpel or electrocautery were included as the selection criteria. Trials wered excluded if they were uncontrolled studies or enrolled patients with immediate reconstruction or breast conserving surgery.

### Data extraction and quality assessment

We extracted the following data from each eligible trial: authors’ names, the journal, year of publication, trial design, operative technique, intraoperative endpoints including operative time, intraoperative blood loss and postoperative endpoints including postoperative drainage, seroma development and wound complications. We contacted to the anthors for the missing or incomplete data. Study quality was assessed using the Jadad score[13].

### Statistical analysis

We examined the potential associations using odds ratios (ORs) with 95% confidence intervals (CIs) for dichotomous endpoints, and standardised mean differences (SMDs)with 95% CIs for continuous endpoints. The pooled ORs and SMDs were considered to be statistically significant if *P*<0.05. Heterogeneity was tested by using the *I* ^2^ statistic and a chi-square test(statistically significant if the *I* ^2^ statistic was greater than 50% or *P*<0.10). When the *P*-value was less than 0.10, the pooled results were calculated using the random-effects model; otherwise, a fixed-effects model was applied. All the statistical analyses were performed using Review Manager V.5.1 (The Cochrane Collaboration, Software Update, Oxford, UK) and stata, version 12.0 (Stata Corporation, College Station, TX).

## Results

### Characteristics of included studies

**Fig 1:**
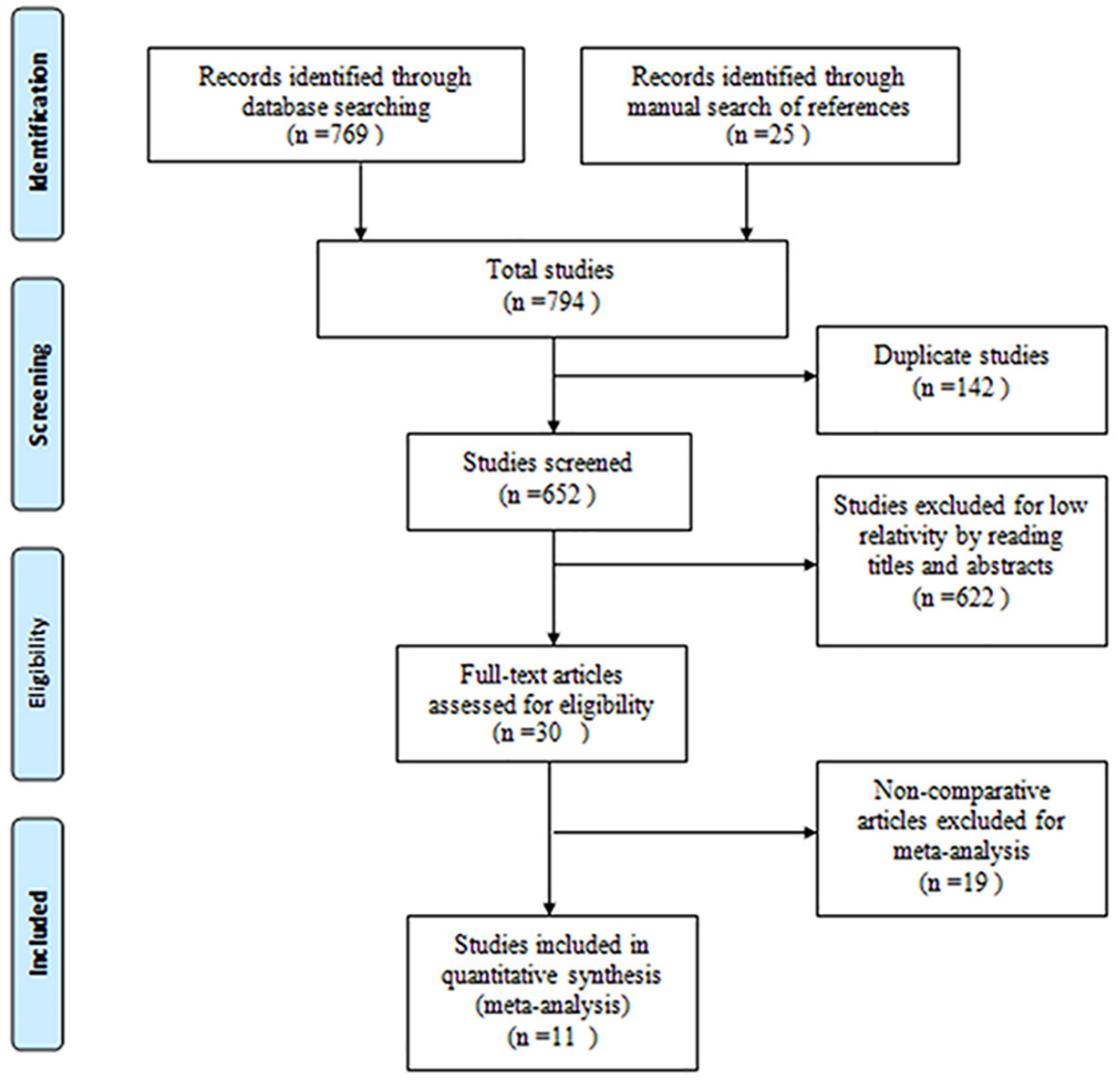
Flow diagram of the study selection process for the meta-analysis.

According to the selection criteria, 794 records were identified by a comprehensive literature search. The study search process was shown in Fig 1. Excluding the reduplicative and uncorrelated studies, A total of 11 studies with 702 patients were included for meta-analysis[7–10,12,14–19]. The characteristics of included studies wered showed in Table 1.

**Table 1:** Characteristics of eligible studies included in this meta-analysis.

Study	Country	Sample size	Study design	Operative technique	Harmonic scalpel type	Electrocautery type	Quality score
Deo et al 2002[7]	India	46	Prospective comparative	MRM	Ultracision	Olympus	N/A
Galatius et al 2003[9]	Denmark	59	Prospective comparative	MRM	Ultracision	Olympus	N/A
Adwani et al 2006[14]	UK	42	Prospective comparative	MRM	Ultracision	Olympus	N/A
Kontos et al 2008[10]	UK	24	RCT	MRM(23) SM(1)	Ultracision	Valleylab Tyco	3
Kozomara et al 2010[15]	Bosnia and Herzegovina	61	RCT	MRM	Ultracision	Olympus	3
Yilmaz et al 2011[8]	Turkey	55	RCT	MRM	Ultracision	Olympus	3
Rohaizak et al 2013[16]	Malaysia	40	RCT	MRM	Ultracision	Valleylab Tyco	2
Gustavo et al 2013[19]	Brazil	95	Prospective comparative	MRM	Ultracision	WEM	N/A
Anlar et al 2013[17]	Turkey	80	RCT	MRM	Ultracision	Olympus	1
Damani et al 2013[18]	Pakistan	50	RCT	MRM	Ultracision	Olympus	1
Salma et al 2014[12]	Pakistan	150	RCT	MRM	Ultracision	Olympus	4

RCT: randomized controlled trials; MRM: modified radical mastectomy; SM: simple mastectomy

### Meta-analysis of the Primary Endpoints

#### Postoperative drainage

**Fig 2:**
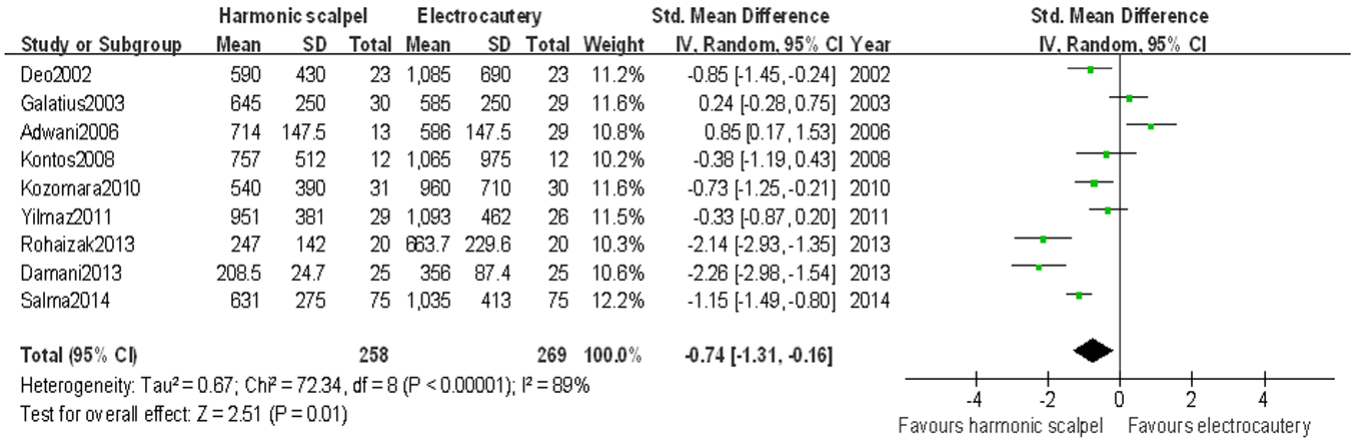
Forest plot for weight mean difference in total postoperative drainage between harmonic scalpel dissection and electrocautery used by random-effect model.

Eleven studies[7–10,12,14–19] referred to the postoperative drainage. Two studies[17,18] wered excluded for the meta-analysis for the missing data of standard deviation. Actually, nine studies[7–10,12,14–16,19] wered included for analysis. The mean drainage volume was 587 mls for ultrasonic dissection group and 825 mls for electrocautery group. Significant difference in total postoperative drainage between harmonic scalpel dissection and standard electrocautery was found (SMD: -0.74 [95%CI: -1.31, -0.16]; *P* < 0.01) (Fig 2). There was significant statistical heterogeneity between the included studies (Chi^2^ = 72.34; *P* < 0.01; *I* ^2^ = 89%).

#### Seroma development

**Fig 3:**
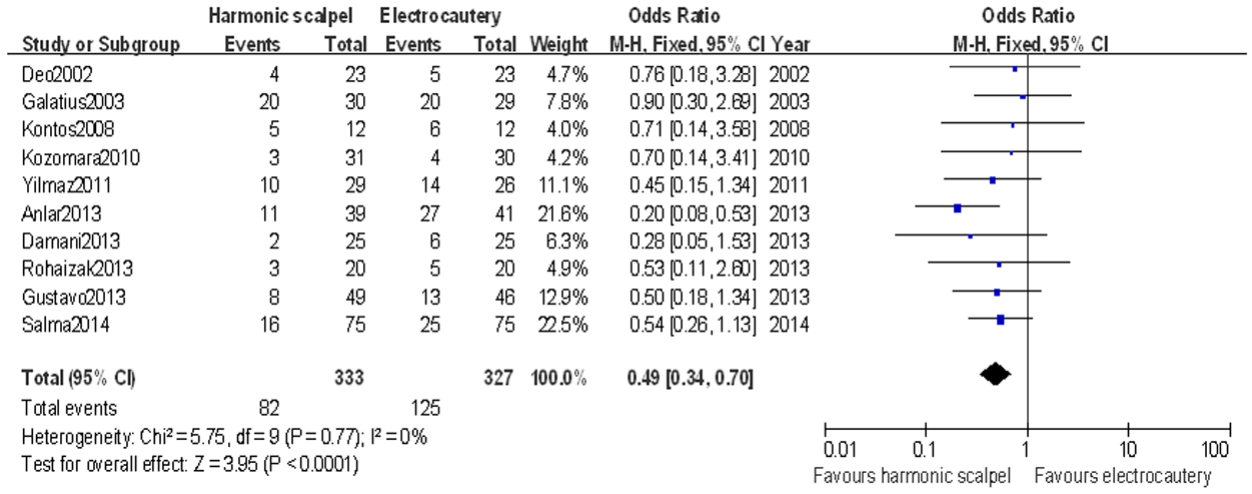
Forest plot for odds ratio of seroma development between harmonic scalpel dissection and electrocautery in mastectomy used by fixed-effect model.

Ten studies[7–10,12,15–19] reported seroma development for the patient after an operation. There were 82 instances of seroma development in the harmonic scalpel group and 125 in the electrocautery group. There was significant difference in terms of seroma development between harmonic scalpel dissection and standard electrocautery by meta-analysis [OR: 0.49 (0.34,0.70); *P* < 0.01] (Fig 3). Low statistical heterogeneity was found between the two groups(Chi^2^ = 5.75; *P* = 0.77; *I* ^2^ = 0%).

#### Intraoperative blood loss

**Fig 4:**
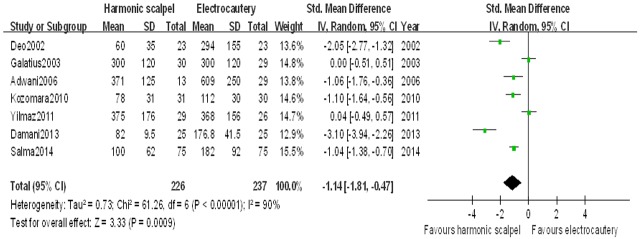
Forest plot for weighted mean difference in intraoperative blood loss between harmonic scalpel dissection and electrocautery in mastectomy used by random-effect model.

Nine studies[7–9,12,14,15,17–19] reported intraoperative blood loss as an endpoint. Two of them[17,19] did not mention the data of standard deviation and we could not get the results. So, a total of seven studies[7–9,12,14,15,18] wered included for this meta-analysis. The mean blood loss volume was 300 mls and 399 mls for harmonic scalpel dissection and electrocautery, respectively. There was significant difference in terms of intraoperative blood loss between the two groups after pooling the included studies (SMD: -1.14 [95%CI: -1.81, -0.47]; *P*< 0.01) (Fig 4). There was significant statistical heterogeneity among the included studies (Chi^2^ = 61.26; *P* < 0.01; *I* ^2^ = 90%).

### Meta- analysis of the Secondly Endpoints

#### Operative time

Ten studies[7–10,12,15–19] reported endpoits of operative time. The mean operative time was 125 min for harmonic scalpel dissection group and 122 min for electrocautery group. There was no statistically significant difference for operative time between the two groups (SMD: 0.04[95%CI: -0.41, 0.50]; *P* = 0.85). There was significant statistical heterogeneity(Chi^2^ = 23.69; *P* < 0.01; *I* ^2^ = 79%).

#### Wound complications

Ten studies[8–10,12,14–19] reported wound complications as an endpoint. There were 43 instances of wound complication in the harmonic scalpel group and 85 in the electrocautery group. There was significant difference in terms of wound complications between harmonic scalpel dissection and standard electrocautery [OR: 0.38 (0.24,0.59); *P* < 0.01]. Low statistical heterogeneity was found between the two groups(Chi^2^ = 11.69; *P* = 0.23; *I* ^2^ = 23%).

### Sensitivity analyses

Sensitivity analyses were performed to explore the source of heterogeneity with respect to the endpoits of postoperative drainage, intraoperative blood loss and operative time by sequential exclusion of individual studies. When we excluded the studies with low quality(The Jadad score < 3) and the study with the largest sample size, the postoperative drainage presented similar pooled results with no evidence of statistical heterogeneity among the remaining studies(Chi^2^ = 1.19; *P* = 0.55; *I* ^2^ = 0%) [8,10,15]. As for intraoperative blood loss, no matter which studies we excluded, the pooled results were similar, but the high statistical heterogeneity was still observed. We surmised the heterogeneity resulted from the surgical operation by different surgeons which led to significant differences in the volume of intraoperative blood loss. After excluding the nonrandomized controlled trials, we got similar pooled results in operative time with low statistical heterogeneity in the remaining studies(Chi^2^ = 10.18; *P* = 0.46; *I* ^2^ = 11%)[8,10,12,15–18].

### Publication bias

Egger’s and Begg’s test were performed to assess the publication bias with the software stata. There was no publication bias for the primary endpoints analyses of postoperative drainage (Egger *P* = 0.97; Begg *P* = 0.75), seroma development (Egger *P* = 0.23; Begg *P* = 0.86), intraoperative blood loss(Egger *P* = 0.32; Begg *P* = 0.23) and for the secondary endpoints analyses (data not shown).

## Discussion

A previous meta-analysis[11] implied that the use of harmonic scalpel in mastectomy showed no advantages as regard to postoperative drainage, seroma development or intraoperative blood loss over standard electrocautery dissection. And also, harmonic scalpel dissection did not provide improved outcomes in operative time and wound complications. However, due to a larger sample size, our meta-analysis demonstrated that harmonic scalpel dissection plays an important role in reducing postoperative drainage, seroma development, intraoperative blood loss and wound complications in MRM for breast cancer. Meanwhile, it did not prolong operating time compared to electrocautery dissection.

The pooled results showed that the use of harmonic scalpel can significantly reduce the total postoperative drainage and seroma development. Seroma development is most commonly implicated with postmastectomy patients with an incidence of 3% to 85%[20]. So that it is thought to be a side effect rather than a complication. Up till now, the reasons as to why seroma forms and its physiological mechanism remain unclear. But some important factors, such as surgical techniques and devices wered reported to have been associated with seroma formation. Patients with MRM were proved to suffer from seroma more easier than those who have breast conservation surgery[21], and immediate breast reconstruction following MRM decreases seroma formation [22]. The use of harmonic scalpel is believed to reduce postoperative drainage and the rate of seroma when compared to that of electrocautery[12,17], for the postulated reasons that thrombosis of subdermal vessels and inadequately sealed lymphatics caused by electrocautery may lead the wound to seroma[23]. Moreover, harmonic scalpel may close vascular and lymphatic channels more precisely by breaking hydrogen bonds to coagulate protein which might be related to reduce inflammatory response and result in preventing seroma development[8]. Other patient and tumor-related factors including body weight[24] and body mass index[25] are associated with seroma formation. It is believed that seroma presents a hindrance in the recovery phase of a patient for the infection and lymphoedema, which impacts continued adjuvant treatments after the operation[20]. Thus, different strategies including the use of fibrin glue[26], somatostatin receptor such as octreotide[27], low vacuum drains in the axilla[28], delaying physiotherapy[29] and shoulder immobilization[30] wered reported to probably reduce seroma development. When it develops, the seroma can be resolved by aspiration or placement of a seroma catheter if a patient has fluid reaccumulation after aspiration[22]. All the strategies mentioned above were not used in the included studies.

With respect to intraoperative blood loss, the previous meta-analysis found a reduction in the mean intraoperative blood loss(236 vs.365 mls) after mastectomy, but the difference was not statistically significant between using harmonic scalpel and electrocautery [11]. However, our pooled analysis revealed that a statistically significant reduction was found in intraoperative blood loss for patients undergoing MRM with the use of harmonic scalpel dissection when compared to electrocautery. The harmonic scalpel has been thought to lower thermal injury to lateral tissues with the vibrating property in animal models[31] and make the haemostasis more sufficient and the dissection more precise[32]. This may present obvious superiority during axillary dissection because there are more branch vessels and important nerves in the dissection plane[33].

The rate of wound complications is decreased by the use of harmonic scalpel in this meta-analysis. Complications other than seromas can not be ignored in breast surgery because they can similarly cause delay of further treatment. The most common complications include flap necrosis, haematoma and wound infection[21]. They are encountered more often in electrocautery dissection for its thermal tissue injury to subdermal vascular plexus and incomplete occlusion of lymphatic channels[12]. Whereas, harmonic scalpel displays advantages in dissection with less thermal tissue injury which can decrease the flap necrosis. What is more,the low thermal condition is not conducive to bacterial growth which leads to the reduced infection rates[10]. The advantages were confirmed by our pooled analysis.

The use of harmonic scalpel in open operation was considered to increase the operating time when compared to electrocautery. But this can be avoided by exposing the surgeons to the new technology prior to the study[16]. The pooled results confirmed that no difference was found with respect to operating time between the two groups. However, what we should pay more attention to is the cost, though it was not included as an outcome in this meta-analysis. The expensive price of harmonic scalpel limits its use in MRM, especially in developing countries. It was reported that the disposable active element of the harmonic scalpel was about 30 times more expensive than the electrocautery spatula[10]. Nevertheless, the benefits of harmonic treatment offset the additional cost by reducing the cost of health care and shortening the time of adjuvant treatment. Moreover, the harmonic scalpel can be used four or five times after sterilization to lower the cost in some low-income countries[12]. More studies are required to evaluate the cost and benefits of the instrument.

Though we included the latest data, the limitations also need to be addressed in this meta-analysis. Some data were excluded from our analysis for the loss of contact for missing data of standard deviation in the original study[17,19], which could cause some bias in the final assessment but was unlikely to change our major conclusions, because the mean values were consistent with our conclusions. The sample size of most of the included studies were small to moderate and some of them were nonrandomized controlled trials with low quality, which might lead to the heterogeneity of the included studies. Therefore, high quality and increased sample size are recommended in further research.

In conclusion, the current meta-analysis indicated that compared to electrocautery, the use of harmonic scalpel can decrease postoperative drainage, seroma development, intraoperative blood loss and wound complications in MRM for breast cancer without increasing operative time. Harmonic scalpel can be recommended as a preferential surgical instrument in MRM. However, further studies with large sample sizes and better study designs are required to confirm our findings.

## Supporting Information

S1 Checklist PRISMA 2009 checklist for the manuscript. (DOC)

S1 File Datasheet for the endpoints between harmonic scalpel and electrocautery in modified radical mastectomy. (XLS)
